# C-952-07. Reducing Small-bore Feeding Tube Complications in Burn Care: A Quality Improvement Project

**DOI:** 10.1093/jbcr/irag033.123

**Published:** 2026-04-06

**Authors:** Rosa de la Cotera, Naureen Sajwani, Sebastian Vrouwe

**Affiliations:** University of Chicago Medicine, Chicago, IL; University of Chicago Burn Center, Chicago, IL; University of Chicago, Chicago, IL

## Abstract

**Introduction:**

Early and aggressive enteral nutrition is an essential component of burn care, commonly delivered via small-bore nasogastric or nasojejunal feeding tube. Unlike other populations where tubes are placed primarily for dysphagia, burn patients require enteral access to meet the excessive metabolic demands during wound healing over weeks to months. Small-bore tubes, however, are susceptible to mechanical complications after an extended insertion time. A retrospective audit of tubes inserted for greater than 30 days (n = 12) between October 2022 through October 2023 showed that 58% (n = 7) experienced a complication including kinking, small aneurysm formation and/or tube breakage. This prompted a quality improvement initiative to develop a guideline for prophylactic tube replacement. Based on a literature review of current evidence-based practice as well as manufacturer’s guidelines, the consensus recommended range for single small-bore tube insertion time was between 30 to 45 days. The need for creation of a unit guideline specifying length of insertion time for single bore tubes in long term burn patients was identified.

**Methods:**

A new guideline specifying length of insertion days for single small-bore feeding tubes was implemented April 2024. The number of insertion days was tracked over a 12-month period. Single small-bore tubes inserted for greater than 30 days were prophylactically replaced and inspected for complication including kinking, small aneurysms and/or breakage. This project was formally determined to be quality improvement, not human subjects research, and was therefore not overseen by the Institutional Review Board, per institutional policy.

**Results:**

Over the 12-month period, seven patients were identified as having a small-bore tube inserted greater than 30 days, meeting guideline requirements for prophylactic replacement. Approaching this time point, the burn team was notified for replacement for patient’s with a continued indication for enteral access. Of the seven patients who met guidelines for replacement, no small-bore tube complications were noted including kinking, small aneurysms, or breakage during post-removal inspection. Following introduction of the new guideline, there was a reduction in small-bore tube mechanical complications from 58% to 0%.

**Conclusions:**

The reduced number of small-bore tube complications following introduction of a guideline for long-term burn patients suggests that prophylactically replacing small-bore tubes between insertion day 30-45 reduces the number of small-bore tube complications.

**Applicability of Research to Practice:**

Consider prophylactic replacement of small-bore feeding tubes present for 30-45 days to reduce the number of mechanical complications.

**Funding for the study:**

N/A.

